# Mineral bone disorder in chronic kidney disease: head-to-head comparison of the 5/6 nephrectomy and adenine models

**DOI:** 10.1186/1471-2369-15-69

**Published:** 2014-05-03

**Authors:** Guaraciaba O Ferrari, Juliana C Ferreira, Raquel T Cavallari, Katia R Neves, Luciene M dos Reis, Wagner V Dominguez, Elizabeth C Oliveira, Fabiana G Graciolli, Jutta Passlick-Deetjen, Vanda Jorgetti, Rosa MA Moysés

**Affiliations:** 1 Nephrology Department, Universidade de São Paulo, Rua Iperoig, 690, apto 121, São Paulo, Brazil; 2 Division of Nephrology, University of Dusseldorf, Dusseldorf, Germany

**Keywords:** Bone disease, Chronic kidney disease, Hyperparathyroidism, Phosphate binder, Vascular calcification

## Abstract

**Background:**

Experimental models are important to the understanding of the pathophysiology of, as well as the effects of therapy on, certain diseases. In the case of chronic kidney disease-mineral bone disorder, there are currently two models that are used in evaluating the disease: 5/6 nephrectomy (Nx) and adenine-induced renal failure (AIRF). However, the two models have never been compared in studies using animals maintained under similar conditions. Therefore, we compared these two models, focusing on the biochemical, bone histomorphometry, and vascular calcification aspects.

**Methods:**

Wistar rats, initially fed identical diets, were divided into two groups: those undergoing 5/6 Nx (5/6Nx group) and those that were switched to an adenine-enriched diet (AIRF group). After 9 weeks, animals were sacrificed, and we conducted biochemical and bone histomorphometry analyses, as well as assessing vascular calcification.

**Results:**

At sacrifice, the mean body weight was higher in the 5/6Nx group than in the AIRF group, as was the mean blood pressure. No differences were seen regarding serum phosphate, ionized calcium, intact parathyroid hormone (PTH), or fibroblast growth factor 23 (FGF23). However, creatinine clearance was lower and fractional excretion of phosphate (FeP) was higher in the AIRF group rats, which also had a more severe form of high-turnover bone disease. Vascular calcification, as evaluated through von Kossa staining, was not observed in any of the animals.

**Conclusions:**

Overt vascular calcification was not seen in either model as applied in this study. Under similar conditions of diet and housing, the AIRF model produces a more severe form of bone disease than does 5/6 Nx. This should be taken into account when the choice is made between these models for use in preclinical studies.

## Background

Chronic kidney disease (CKD) is a worldwide health problem, commonly associated with high morbidity and mortality. One common and challenging complication is CKD-mineral bone disorder (CKD-MBD), defined by abnormalities in mineral and hormone metabolism, as well as bone histological changes, with or without soft tissue calcification [1]. However, many pathophysiological aspects of CKD-MBD are still not completely understood. As a result, new therapeutic strategies, although needed, are scarce. In addition, clinical trials that could improve our understanding of CKD-MBD and the potential benefits or hazards of therapeutic interventions are extremely difficult and costly to conduct. In this regard, animal models could be a strategic option, providing the opportunity to examine the basic aspects of this disease, and its treatment.

Until recently, 5/6 nephrectomy (Nx), first described at the beginning of the 20th century [2], was the most common animal model used in preclinical studies evaluating CKD-MBD. However, the 5/6Nx model has several drawbacks: it requires surgical skill; it has a high mortality rate; and pronounced MBD takes several weeks to develop [3]. During the last few years, a model originally suggested in the 1980s [4], based on the nephrotoxicity provoked by adenine, has been also employed in this setting. In that model, CKD is induced by administration of adenine in diet or solution, thereby precluding the need for surgery. Authors who have employed the adenine model to evaluate CKD-MBD have reported that it provokes a more severe form of the disease, with impressive bone alterations, as well as significant VC [5,6]. Nevertheless, it is difficult to compare the results obtained with these two models, because the dietary protocol employed has not been standardized (neither in terms of the amount and duration of adenine administration, nor in terms of the dietary concentrations of calcium, phosphate, and protein), as well as because the observation period and therapeutic strategies have varied across studies. Therefore, the exact differences between the 5/6Nx and adenine models, as well as which would be the best model to evaluate a specific aspect of bone disease, remain unclear. To answer these questions, we decided to compare both models under identical conditions, in terms of animal species, diet, and duration of CKD.

## Methods

### Ethics statement

All experimental procedures were approved by and conducted in accordance with the guidelines of the Standing Committee on Animal Research of the Universidade de São Paulo School of Medicine (Protocol no. 0447/08).

### Experimental protocol

Thirty five male Wistar rats, weighing 300–350 g, were obtained from our local breeding colony. They were housed in individual cages in a temperature- and humidity-controlled environment (25°C at 25% humidity), on a 12/12-h light/dark cycle, and fed a standard powder diet containing 1.17% calcium, 0.93% phosphate, 19.3% protein, and 4.2 IU/g vitamin D_3_ (LabDiet^®^ 5C3Y; PMI Nutrition International, St. Louis, MO, USA), with ad libitum access to water. After 1 week of acclimatization, rats were allocated to be submitted to 5/6Nx, which consisted of removal of the right kidney and infarction of approximately 2/3 of the left kidney, or to start an adenine-enriched diet (LabDiet^®^ 5 K52 plus 0.75% and 0.50% adenine; PMI Nutrition International). A normal renal function Control group was also allocated. The Control and the 5/6Nx groups rats were fed the standard diet throughout the study. For the AIRF group, the adenine concentration was 0.75% in the first two weeks and 0.50% in the following two weeks. Thereafter, the AIRF group rats were fed the standard diet [7]. A pair-feeding protocol was used. On a weekly basis, the rats were weighed and blood pressure was measured by tail cuff plethysmography. At 9 weeks after the surgical procedure and the start of adenine administration, rats were anesthetized and sacrificed by exsanguination via aortic puncture.

We injected each rat intraperitoneally with 25 mg/kg oxytetracycline (Terramycin^®^; Pfizer Animal Health, New York, NY, USA), a fluorescent marker of bone, on days 51, 52, 58 and 59 of the protocol, for dynamic evaluation of bone histomorphometry. We also collected 24-h urine samples, 1 day before the sacrifice, in order to quantify urine output and perform biochemical analysis.

Serum samples were collected at the time of sacrifice by aortic puncture and stored at -20° for subsequent biochemical evaluation. The heart was excised, and the left ventricle was dissected for weighing and determination of the left ventricle weight normalized to body weight (LVW/BW). Thoracic and abdominal aortas were removed in order to evaluate VC, by von Kossa staining and calcium content quantification, respectively. Femurs were removed for bone histomorphometry.

### Biochemical analysis

In serum samples, we measured creatinine and phosphate by colorimetric assay (Labtest, Lagoa Santa, Brazil); ionized calcium was measured with an ion-selective analyzer (AVL-9140; AVL Scientific Corporation, Roswell, GA, USA); the PTH level was determined by enzyme-linked immunosorbent assay (ELISA kit; Immutopics, San Clemente, CA, USA), as was the level of FGF23 (ELISA kit; Kainos Laboratories, Tokyo, Japan); calcitriol was quantified by radioimmunoassay using a pool of samples. In urine samples, we quantified creatinine, phosphate, and calcium by colorimetric assay (Cobas Amplicor; Roche Diagnostics, Indianapolis, IN, USA).

### Bone histomorphometry

The left femur of each rat was removed, dissected free of soft tissue, immersed in 70% ethanol, and processed as previously described [8]. Using a Jung K microtome (Reichert Jung, Heidelberg, West Germany), we cut distal femurs into sections of 5 μm and 10 μm thickness. The 5-μm sections were stained with 0.1% toluidine blue, pH 6.4, and we examined at least two nonconsecutive sections per sample.

Structural, static, and dynamic parameters of bone formation and resorption were measured at the distal metaphysis (magnification, ×250), at 195 μm from the epiphyseal growth plate, in a total of 30 fields, using a semi-automatic image analyzer (Osteomeasure; Osteometrics, Atlanta, GA, USA). Structural parameters included trabecular thickness (in μm), trabecular separation (in μm), and trabecular number (in trabeculae/mm). The indices of static formation included the proportions of trabecular bone volume and osteoid volume to total bone volume (both in %); osteoid thickness (in μm); and osteoid/osteoblast surfaces (both in % of bone surface). The indices of static resorption included eroded surface and osteoclast surface (both in % of bone surface). The mineral apposition rate, determined by measuring the distance between the two oxytetracycline labels and dividing it by the time elapsed between the two oxytetracycline administrations, is expressed in micrometers per day. Mineralization lag time is expressed in days. Determination of the proportion of double oxytetracycline-labeled surface to total trabecular surface (in %) and of the bone formation rate completed the dynamic evaluation. Results are also described according to the turnover-mineralization-volume (TMV) classification [9]. Histomorphometric indices were reported using nomenclature recommended by the American Society of Bone and Mineral Research [10].

### Vascular calcification

Cross-sections of the thoracic aorta were embedded in paraffin, sectioned at 4 μm, and stained with von Kossa. For the analysis of calcium concentration, the abdominal aorta was solubilized in 0.6 N HCl (40 μL HCl/g tissue) for 48 h, after which it was dried and weighed. The sample was then centrifuged, and the calcium content was measured in the supernatant (Calcium Reagent Set; Pointe Scientific, Canton, MI, USA).

### Statistical analysis

Results are presented as mean ± SD or median (min-max). Comparisons among groups were made using Anova. Graph Pad Prism version 3.03 (GraphPad Software, Inc., San Diego, CA, USA) were used. Values with *P <* 0.05 were considered as statistically significant.

## Results

### Study sample

The final study sample comprised 30 rats: 10 in the 5/6Nx group; 10 in AIRF group; and, 10 in Control group. Although 15 rats were submitted to 5/6 Nx, three died (on post-procedure days 3, 7, and 50, respectively) and two others were excluded from the final analysis because of the severity of renal failure (serum creatinine levels of 6.7 and 3.5 mg/dl, respectively). There were no deaths in the AIRF group. As shown in Table 1, despite the use of a pair-feeding protocol, the mean body weight at sacrifice was lower in the AIRF group. Mean urine output was significantly higher in the AIRF group, whereas mean blood pressure was higher in the 5/6Nx group. No differences were seen regarding the LVW/BW.

**Table 1:** **Characteristics of the rats evaluated**^**1**^

**Variable**	**Control**	**AIRF**	**5/6Nx**
	**(n = 10)**	**(n = 10)**	**(n = 10)**
Initial body weight (g)	324.4 ± 37.4	307.1 ± 30.8	307.9 ± 19.9
Final body weight (g)	366.1 ± 18.7	312.0 ± 17.2^a^	385.4 ± 59.8^a,b^
Food intake (g/day)	17.5 ± 1.4	16.8 ± 1.9	18.9 ± 0.7^b^
Urine output (ml)	11.4 ± 5.4	53.7 ± 11.2^a^	22.7 ± 6.7^a,b^
TCP-BP (mmHg)	119.7 ± 7.3	154.4 ± 20.4^a^	220.0 ± 19.5^a,b^
LVW/100 g BW	0.18 ± 0.01	0.21 ± 0.02^a^	0.21 ± 0.05

^1^Data expressed as mean ± SD.

TCP-BP, Blood pressure, as measured by tail cuff plethysmography.

Initial, Before Nx5/6 and adenine diet protocol.

Final, At the sacrifice.

^a^p < 0.05 vs. Control; ^b^p < 0.05 vs. AIRF.

### Biochemical parameters

Creatinine levels tended to be higher in the AIRF group than in the 5/6Nx group, whereas a significant difference regarding creatinine clearance was seen between uremic groups (Table 2). The two groups were comparable in terms ionized calcium, phosphate, PTH, and FGF23. However, despite the similar serum phosphate levels, FeP was higher in the AIRF group. In addition, calcitriol levels were significantly lower in the 5/6Nx group.

**Table 2:** Biochemical data at the sacrifice

**Variable**	**Control (n = 10)**	**AIRF (n = 10)**	**5/6Nx (n = 10)**
Serum Cr (mg/dl)	0.6 ± 0.1	1.6 ± 0.3^a^	1.3 ± 0.6^a^
Creat Clear (ml/min)	2.55 ± 0.74	0.67 ± 0.25^a^	1.1 ± 0.46^a,b^
Serum iCa (mmol/L)	1.2 ± 0.1	1.1 ± 0.1	1.1 ± 0.1
uCa (mg/dl)	14 (8–17)	6 (4–8)	11 (5–50)
Serum P (mg/dl)	5.6 ± 0.9	7.4 ± 0.7^a^	6.7 ± 1.4
FeP (%)	9.1 ± 4.8	21.2 ± 11.0^a^	7.6 ± 4.6^b^
iPTH (pg/ml)	108 (47–315)	681 (163–1,604)^a^	572 (198–4,199)^a^
FGF23 (pg/ml)	157 (107–310)	784 (418–14.476)^a^	893 (649–2.609)^a^
Calcitriol (pg/ml)*	69.2 ± 17.3	88.0 ± 16.6	35.1 ± 20.0^b^

Data expressed as mean ± SD or median (min-max).

Creat Clear, Creatinine clearance; Serum iCa, Serum ionized calcium; uCa, Urinary calcium; Serum P, Serum phosphate. *Calcitriol was measured in pooled sera.

P < 0.05 ^a^vs Control; ^b^vs AIRF.

### Bone histomorphometry

Bone histomorphometry revealed that the bone formation rate was significantly higher in the AIRF group rats than in the 5/6Nx group rats. Other parameters of bone formation, such as osteoid and osteoblast surfaces, as well as resorption and osteoclast surfaces, were also significantly higher in the AIRF group. The mineralization lag time was higher in AIRF group. We also observed no significant differences between the two groups in terms of trabecular bone volume. However, bone microarchitecture was altered in the AIRF group animals, as evidenced by a lower trabecular number and greater trabecular separation. Fibrosis was observed in only one AIRF rat (Table 3).

**Table 3:** Bone histomorphometry data

**Parameter**	**Control**	**AIRF**	**5/6Nx**
	**(n = 10)**	**(n = 10)**	**(n = 10)**
BV/TV (%)	25.0 ± 4.8	31.1 ± 6.7	30.3 ± 3.7
TbN (n/mm)	3.7 ± 0.6	3.3 ± 0.7	4.7 ± 0.5^a,b^
Tb.SP (μm)	208.8 ± 48.2	223.6 ± 95.6	147.6 ± 26.5^b^
Tb.Th (μm)	67.1 ± 5.5	93.4 ± 8.8^a^	63.8 ± 4.0^b^
ObS/BS (%)	5.4 ± 3.0	25.3 ± 6.2^a^	5.8 ± 2.3^b^
OV/BV (%)	0.45(0.14-0.56)	1.46(0.54-3.78)^a^	0.28(0.20-0.77)^b^
O.Th (μm)	1.7 ± 0.4	2.6 ± 1.3	1.5 ± 0.2^b^
OS/BS (%)	1.6(1.2-2.1)	30.3(18.8-40.5)^a^	6.1(5.1-13.9)^b^
OcS/BS (%)	1.9 ± 1.1	7.4 ± 2.8^a^	3.2 ± 1.5^b^
ES/BS (%)	9.1 ± 1.9	24.5 ± 6.3^a^	12.3 ± 5.1^b^
MS/BS (%)	6.0(3.7-9.1)	10.3(7.1-14.3)	2.4(0.8-5.3)^a,b^
MAR (μm/day)	0.8 ± 0.4	1.0 ± 0.7	0.8 ± 0.2^b^
BFR (μm^3^ /μm^2^ per day)	0.03(0.01-0.05)	0.14(0.01-0.22)	0.02(0.01-0.03)^b^
MLT (d)	3.6(1.6-8.1)	8.4(1.4-136.0)^a^	4.6(3.4-14.8)^b^

Data expressed as mean ± SD or median (min-max).

BV/TV, Bone volume/trabecular volume; TbN, Trabecular number; Tb.SP, Trabecular separation; TbTh, Trabecular thickness; Obs/BS, Osteoblast surface/bone surface; OV/BV, Osteoid volume/bone volume; O.Th, Osteoid thickness; OS/BS, Osteoid surface/bone surface; OcS/BS, Osteoclast surface/bone surface; ES/BS, Eroded surface/bone surface; MS/BS, Mineralization surface/bone surface; MAR, Mineral apposition rate; BFR, Bone formation rate; MLT, Mineralization lag time.

^a^p < 0.05 vs. Control; ^b^vs. AIRF.

### Vascular calcification

Von Kossa staining was negative in both groups. However, calcium content in the abdominal aorta was significantly greater in the 5/6Nx group (Table 4).

**Table 4:** Vascular calcification

**Parameter**	**Control**	**AIRF**	**5/6Nx**
AAC (μmol/mg)	9.6 (8.6-10.6)	7.0 (4.7-11.4)	15.1(13.3-17.9)^a,b^

Data expressed as median (min-max).

AAC, Abdominal aortic calcium.

^a^p < 0.05 vs. Control; ^b^vs. AIRF.

## Discussion

To our knowledge, this was the first study to compare, under similar conditions, the most widely used animal models of CKD-MBD. We showed that, in rats fed identical diets and observed for the same length of time, renal dysfunction and bone disease are more severe when the AIRF model is employed.

Initially described in 1932, 5/6Nx (ligation of the renal artery branches, electrocauterization of the renal cortex or removal of the left kidney poles and right kidney nephrectomy) reduces the effective mass of the kidneys [11]. The effectiveness of this model is dependent upon the anatomy of individual animals and the experience of the researcher, leading to different levels of CKD [12]. According to classical descriptions, animals submitted to 5/6Nx develop hyperphosphatemia, reduced calcitriol levels, and hyperparathyroidism, as well as VC and high-turnover bone disease [3,13-15]. VC is typically seen at approximately 12 weeks after the surgical procedure unless the animals are fed with a high-phosphate diet or calcitriol is administered to them [16]. Of the 15 rats initially included in our 5/6Nx group, three died. This could be attributed to the surgical procedure itself or to greater renal disease severity. Given that two additional animals were excluded from the final analysis because they had extremely high serum creatinine levels, it is clear that, when the 5/6Nx model is employed, such losses must be taken into account and a higher number of rats should be included in the original sample. Considering the potential expenses involved, this could be viewed as a disadvantage of the 5/6 Nx model in comparison with the AIRF model.

The adenine model of nephrotoxicity was first described in 1982, when Yokozawa et al. observed that 2.8-dihydroxyadenine, a metabolite of adenine, precipitated as crystals in renal tubules, causing rats fed an adenine-rich diet to develop uremia [4]. Since then, adenine-enriched diets or solutions of adenine, in various concentrations, have been used by many authors for varying periods of time and for different purposes. In 2003, Katsumata et al. used this model, feeding rats a 0.75% adenine diet for 3 weeks in order to study the effects of sevelamer [5]. In 2006, Tamagaki et al. analyzed the bone and metabolic characteristics of AIRF in rats after 2, 4, and 6 weeks on a 0.75% adenine diet and showed that VC develops only after 4 weeks, whereas incipient bone disease was seen at 2 weeks, leading to a severe skeletal impairment after 6 weeks [6]. In that same year, Nagano et al. published the results of an experiment involving a different dose of adenine (0.75% for 2 weeks followed by 0.50% for 2 weeks) and showed that the rats developed renal failure, as well as hyperphosphatemia, secondary hyperparathyroidism, and reduced calcitriol levels [7]. Subsequent studies involved different adenine concentrations and periods of administration [5-7,12,14,17,18]. So, it seems clear that the degree of renal dysfunction, as well as the extent of bone disease and VC, is totally dependent on the amount of adenine administered and the duration of administration.

One important element in the construction of an experimental model of CKD is the amount of calcium and phosphate administered in the diet, as well as the source of phosphate (grain or casein). The bioavailability of phosphate is greater for diets based on casein (cow-milk protein) than for those based on grain [19]. In addition, the phosphate load should be quantified by measuring the FeP, given that serum phosphate might not increase if parathyroid function is preserved since PTH increases renal phosphate excretion. Therefore, in the present study, we designed an experiment in which we tested the 5/6Nx and AIRF under identical conditions in terms of the calcium, phosphate (from grain), and protein content of the diet.

We found that the AIRF group rats had lower weight, despite the pair-feeding protocol, as has been reported previously [6]. This has been attributed to the poor palatability of adenine, as well as to the uremia and polyuria caused by tubular disease, which in addition to the ingestion of a minimal amount of water, could lead to dehydration and weight loss [20]. This finding should be interpreted as a limitation of AIRF.

The 5/6Nx model induces a progressive glomerular sclerosis, whereas the adenine model promotes intratubular crystal precipitation and interstitial nephritis. Based on this, we would expect to find proteinuria, hypertension and cardiac hypertrophy in the first model, whereas acidosis and more severe bone lesions would be seen in the second model. In our experiment, the LVW/BW was similar between the two groups, despite the higher blood pressure observed in the 5/6Nx group. This was probably because the AIRF group rats showed greater uremia, which is also a risk factor for ventricular hypertrophy (in fact, we found a positive correlation between serum creatinine and LVW/BW; R = 0.69, p < 0.05). Therefore, the 5/6Nx model is indicated for studies evaluating ventricular hypertrophy associated with hypertension, whereas the adenine model would be more appropriate for the study of other variables that could lead to ventricular hypertrophy.

We found the two groups to be comparable in terms of serum and urinary calcium, as well as serum phosphate. However, FeP was found to be significantly higher in the AIRF group, which could be explained by the increased bone turnover or by tubulointerstitial nephropathy, with direct tubular damage leading to phosphaturia. In favor of the latter theory, we found no statistically significant correlation between FeP and the two major phosphaturic hormones, PTH and FGF23, nor between FeP and bone resorption parameters (data not shown). However, a direct effect of adenine on the tubular excretion of phosphate has never been shown. Our data also show that there were no significant differences between the two models in terms of PTH or FGF23. Various authors have reported an extreme increase in PTH levels in the adenine model. However, some [6] administered adenine for a longer period time, whereas others measured PTH using assays that were different from ours, impeding comparisons across studies. In addition, as previously mentioned, none of the previous studies compared the adenine model to 5/6Nx model in a single experiment. Surprisingly, we found higher calcitriol levels in AIRF than in Nx group. However, calcitriol levels in AIRF were not different from Control group, results that are similar to those were described by Damment et. al, who found no significant differences in calcitriol levels between normal renal function and chronic renal failure, adenine-treated rats [17]. The reasons for this unexpected finding could be the combination of non-significant higher PTH levels and lower FGF-23 in adenine-treated rats or a technical issue with calcitriol measurements, which are usually done in pools of sera, due to the significant amount of serum that is necessary for this procedure or even an increased calcitriol synthesis in the interstitial renal granulomas that are usually seen around adenine crystals [21].

The bone effects of 5/6Nx and AIRF are distinct, confirming previous findings that the natural history of CKD-MBD could be also dependent on the model that was chosen to perform the experiments [22,23]. In our study, we found a high-turnover bone disease in AIRF. Notable among our histomorphometric findings are the bone formation rate and the proportion of osteoid surface, which were 5 times higher in the AIRF group rats than in the 5/6Nx group rats. We also found a positive correlation between serum creatinine and bone formation rate (R = 0.59; p < 0.05), showing that worse renal function leads to higher bone turnover. Nevertheless, osteoblasts were apparently unable to increase bone matrix deposition to the same degree, probably because this process is not only cell-mediated but also physicochemical, as AIRF caused greater phosphate loss in urine, probably impairing mineralization. In addition, accumulating evidence suggests that extracellular nucleotides, signaling through P2 purinoceptors, play an important role in modulating bone cell function. Adenosine triphosphate and other nucleotides can stimulate the formation and resorptive activity of osteoclasts, as well as inhibiting bone mineralization by osteoblasts [24]. Because adenosine is derived from adenine, a purine base, we cannot rule out the hypothesis that high doses of adenine lead to an increase in adenosine, which might have direct effects on the function of osteoblasts and osteoclasts. However, we did not observe any crystal in bone marrow in our samples. Another possibility is that the lower calcitriol levels described in some adenine animal studies could be responsible for the observed increase in osteoid parameters; however, in the present study, calcitriol levels were significantly higher in the AIRF group than in the 5/6Nx group. Although metabolic acidosis could also influence bone findings in the adenine model, we did not measure whole blood pH, which could be viewed as a limitation of our study. However, a recent study employing the adenine model, in which serum creatinine at the end of the study was similar to that described in our experiment, showed levels of pH and CO_2_ that could not explain the magnitude of the bone findings, making it less probable that metabolic acidosis is a relevant factor [17]. Nevertheless, despite the fact that the cause of the increased amount of osteoid observed in the adenine model remains unknown, this finding should be kept in mind when choosing a model to evaluate the bone effects of certain drugs, such as phosphate binders. The administration of these drugs in the adenine model of CKD could increase osteoid parameters, as previously shown by Neven et al. [18].

We found it surprising that the von Kossa staining was negative in both groups and that the calcium concentration in the abdominal aorta was higher in the 5/6Nx group. This would seem to be in disagreement with the current concept that the AIRF frequently leads to VC. However, our animals received less adenine than did those described in the study conducted by Tamagaki et al. [6], which treated animals for 6 weeks with 0.75% adenine and observed massive VC. Therefore, it is likely that our protocol of adenine administration was insufficient to provoke the expected VC. In relation to 5/6Nx, other studies employing this model have shown that, in nephrectomized rats, VC is not detected through von Kossa staining in normal conditions [3]. Therefore, it is understandable that the von Kossa staining was negative in the 5/6Nx group rats, since our study had a duration time of 9 weeks. Another question is why the 5/6Nx group rats presented higher calcium concentration in the abdominal aorta than did the AIRF group rats. One hypothesis is that the duration of CKD could be different in the two models. Another possibility is that the high blood pressure in 5/6Nx model could lead to a higher calcium deposition. Therefore, if one plans to evaluate massive VC, experimental animals must be fed higher concentrated adenine-diet for a longer period of time or must be observed after several months in the 5/6Nx model [25].

Our study presents several limitations: we did not measure biochemical variables or performed histomorphometric analysis at intermediate time points; we did not evaluate different concentrations of adenine in the diet; we could not separate the effects of PTH, FGF-23 or calcitriol, or kidney function in each of these models. In addition, the duration of our experiment mimics approximately 4–5 years in human disease and it is known that CKD-MBD has an insidious course of many years. However, we were able to demonstrate for the first time in a head-to-head comparison, that animals present different forms of CKD depending on the model that is chosen. In face of our results, 5/6 Nx model should be employed to study the effects of intervention on blood pressure, on kidney disease progression, on serum levels of PTH, FGF-23 and calcitriol or on mild bone lesions of secondary hyperparathyroidism. It should also be used in studies with longer observation period. On the other hand, AIRF model should be used to evaluate ventricular hypertrophy not related to hypertension or volume overload. The direct effect of adenine crystals on renal tubules and interstitium impairs its use on studies of effects of CKD-MBD therapy on CKD progression. Long term studies are also difficult with this model because there is a progressive undernutrition and severe renal disease, unless lower concentrations of adenine are employed.

## Conclusion

In summary, our study shows the peculiarities of the adenine and 5/6Nx models in terms of the CKD-MBD induced. Its main strength is that, for the first time, 5/6Nx and adenine models were compared under similar conditions of housing, feeding, and observation period. Using the same experimental protocol for both models, we found that the adenine model provokes massive phosphaturia and a more severe form of bone disease, although we observed no significant differences between the two models in terms of VC. Nevertheless, the differences identified should be taken into account when a specific model is selected in order to evaluate the effects of CKD-MBD therapies.

## Competing interests

The authors declare that they have no competing interests.

## Authors’ contributions

GOF: performed the experiments, analyzed the data, wrote the manuscript; JCF: performed the experiments; RTC: performed the experiments; KRN: conceived and designed the experiments, performed the experiments; LMR: performed the experiments; WVD: performed the experiments; ECO: performed the experiments; FGG: performed the experiments; JPD: conceived and designed the experiments, VJ: conceived and designed the experiments; RMAM: conceived and designed the experiments, performed the experiments, analyzed the data, wrote the manuscript. All authors read and approved the final manuscript.

## Pre-publication history

The pre-publication history for this paper can be accessed here:

http://www.biomedcentral.com/1471-2369/15/69/prepub
